# Extracorporeal membrane oxygenation line-associated complications: in vitro testing of cyanoacrylate tissue adhesive and securement devices to prevent infection and dislodgement

**DOI:** 10.1186/s40635-018-0171-8

**Published:** 2018-03-12

**Authors:** Taressa Bull, Amanda Corley, Danielle J. Smyth, David J. McMillan, Kimble R. Dunster, John F. Fraser

**Affiliations:** 1 Critical Care Research Group, The Prince Charles Hospital and University of Queensland, Level 3 Clinical Sciences Building, Rode Rd, Chermside, 4032 Queensland Australia; 2 Bacterial Pathogenesis Laboratory, Queensland Institute of Medical Research, Herston Rd, Herston, 4006 Queensland Australia; 3 Inflammation and Healing Research Cluster, Faculty of Science, Health, Education and Engineering, University of the Sunshine Coast, 90 Sippy Downs Dr, Sippy Downs, 4556 Queensland Australia

**Keywords:** Extracorporeal membrane oxygenation, Cyanoacrylate, Tissue adhesives, Securement, Catheter-related infections

## Abstract

**Background:**

Extracorporeal membrane oxygenation (ECMO) delivers cardiac and/or respiratory support to critically ill patients who have failed conventional medical therapies. If the large-bore cannulas used to deliver ECMO become infected or dislodged, the patient consequences can be catastrophic. ECMO cannula-related infection has been reported to be double the rate of other vascular devices (7.1 vs 3.4 episodes/1000 ECMO days respectively). The aim of this study was to assess the ability of cyanoacrylate tissue adhesive (TA) to inhibit bacterial growth at the ECMO cannulation site, and the effectiveness of TA and securement devices in securing ECMO cannulas and tubing.

**Methods:**

This in vitro study tested the (1) antimicrobial qualities of TA against standard transparent dressing with ECMO cannula; (2) chemical compatibility between cannula, TA and removal agent; (3) pull-out strength of transparent dressing and TA at the cannula insertion site; and (4) pull-out strength of adhesive bandage and commercial sutureless securement devices (SSDs) on circuit tubing. Fisher’s exact test was used to evaluate differences in bacterial growth observed between the transparent dressing and TA groups. Data from mechanical testing were analysed using one-way ANOVA, followed by Tukey’s multiple comparison test or *t* test as appropriate. Statistical significance was defined as *p* < 0.05.

**Results:**

No bacterial growth occurred under TA-covered cannulas compared with transparent dressing-covered cannulas (*p* = 0.002). Compared to plates lacking TA or transparent dressing, growth was observed at the insertion point and under the dressing in the transparent dressing group; however, no growth was observed in the TA group (*p* = 0.019). TA did not weaken the cannulas; however, the TA removal agent did after 60 min of exposure, compared with control (*p* < 0.01). Compared with transparent dressing, TA increased the pull-out force required for cannula dislodgement from the insertion point (*p* < 0.0001). SSDs significantly increased the force required to remove the tubing from the fixation points compared with adhesive bandage (*p* < 0.01).

**Conclusions:**

Our findings suggest that the combined use of TA at the cannula insertion site with a commercial device for tubing securement could provide an effective bedside strategy to prevent or minimise infection and line dislodgement.

## Background

Extracorporeal membrane oxygenation (ECMO) is a form of cardiopulmonary bypass used to support patients with severe cardiac and/or respiratory failure who have failed maximal conventional medical management [1, 2]. Nosocomial infection is one of the most frequent and serious complications of ECMO [3, 4]. It is often difficult to treat [5] and has been reported in up to 64% of ECMO patients [4]. Infections acquired whilst on ECMO are associated with increased durations of ECMO support and ventilatory support [3, 4, 6–9], and longer intensive care unit and hospital lengths of stay [4, 10]. Poorer prognosis has also been reported in the paediatric population [11]. ECMO patients are a high-risk group for nosocomial bloodstream infections, which is partly attributable to the large-bore ECMO cannulas and multiple standard invasive devices needed to manage these critically ill patients [3, 10]. Bloodstream infection rates on ECMO range from 3 to 19 per 1000 ECMO days [3, 4, 7, 8, 10]. Less frequently reported but equally important is the risk for localised cannula infection. ECMO cannula-associated infection prevalence ranges between 10 and 17% [4, 12, 13], with double the rate of infection associated with ECMO cannulas compared with other vascular devices (7.1 vs 3.4 episodes/1000 ECMO days respectively) [4].

ECMO lines (i.e. cannulas and associated tubing) are key components of the extracorporeal circuit but are associated with a large number of the causes of morbidity. Effective securement of these lines to a surface, such as the patient’s limb, is necessary to prevent line movement, malposition or kinking that can precipitate obstruction, low circuit flow and poor venous return, all of which compromise the effectiveness of support [14]. Additionally, incorrectly positioned cannulas lead to turbulent blood flow and cannula ‘kicking’, thereby increasing the risk of haemolysis and renal dysfunction [15]. More serious complications involve partial or complete cannula dislodgement with resultant rapid loss of ECMO support, air entrainment or catastrophic bleeding [16]. Line movement may also lead to local infection and bloodstream infection by facilitating cannula micromotion with the entry of skin-borne organisms through the insertion site or along the cannula into the bloodstream, as occurs with smaller intravascular catheters [17, 18]. Routine removal or re-siting of contaminated ECMO lines is not feasible and may be dangerous to the patient [19], and the clinician is sometimes forced into the scenario of premature termination of ECMO support due to cannula site infection. Therefore, adequate securement is paramount in preventing line complications and maintaining ECMO support for its intended duration.

Despite the universal acceptance of the importance of ECMO cannula securement, there are currently no data on best practice for the effective dressing and securement of ECMO cannulas. Currently, in many centres, ECMO cannula sites are covered with transparent dressings to protect the site from extrinsic contamination. Securement methods to stabilise ECMO lines and prevent gross movement include tapes, adhesive bandages and sutures, although practice lacks standardisation and no evidence-based recommendations exist. Commercial sutureless securement devices (SSDs) provide a more contemporary approach to ECMO line management at the bedside. However, there is mixed evidence regarding their efficacy in securing intravascular catheters [20, 21] and SSDs have never been tested with ECMO cannulas. Medical-grade skin glue (cyanoacrylate), also known as tissue adhesive (TA), has undergone recent investigation as a novel product for securing invasive devices [18, 20, 22–27] and reducing associated infection risk [25, 28, 29]. TA applications for improved intravascular catheter securement compared with transparent dressing include epidural [26], central venous [27], peripheral venous [22, 25] and arterial catheters [23, 24]. TA also has antimicrobial properties and in vitro testing small-gauge catheters has shown inhibition of Gram-positive organisms, suggesting its potential to reduce the incidence of catheter-associated infection [25, 29].

To date, there has been no published research on ECMO line securement, and TA is untested in large-bore intravascular devices such as ECMO cannulas. We hypothesised that TA could improve ECMO cannula securement and inhibit bacteria at the cannula site. Given the imperative to prevent ECMO line complications, we tested in vitro the safety and feasibility of TA to prevent bacterial colonisation of cannula sites and for cannula site securement when compared with transparent dressing. We additionally compared adhesive bandage and two commercial SSDs for sutureless ECMO tubing securement.

## Methods

This study comprised four parts: (1) assessment of microbiological qualities of TA against standard transparent dressing at the ECMO cannula insertion site; (2) assessment of chemical compatibility between cannula, TA and a TA removal agent; (3) comparison of pull-out strength between transparent dressing and TA at the cannula insertion site; and (4) comparison of pull-out strength between adhesive bandage securement and commercial sutureless securement devices on ECMO circuit tubing.

### Microbiological tests

The ability of TA (Histoacryl; Butyl-cyanoacrylate; B. Braun, Tuttlingen, Germany) and transparent dressing (Opsite Incise Drape; Smith & Nephew, London, UK) to inhibit *Staphylococcus epidermidis* (*S. epidermidis* RB02) migration and colonisation of ECMO cannulas was investigated using methods previously described [25]. The *S. epidermidis* RB02 used was an isolate from a clinical specimen previously used in similar assays by our team [25]. Briefly, sterile 23F Bio-Medicus femoral (Medtronic Inc., Minneapolis, MN) wirewound polyurethane venous cannulas were cut into 5-cm lengths, and each length was aseptically inserted at an angle approximately 30° into an agar plate containing the pH indicator bromocresol purple [30]. The cannula plates were separated into three groups, each group comprising of six replicate plates (*n* = 6). Group 1 had transparent dressing (Opsite, 7 cm × 7 cm) covering the insertion point; group 2 had TA (Histoacryl, 0.5 mL) applied at the insertion point and allowed to dry; group 3 was treated with both TA and transparent dressing. TA and transparent dressings were applied according to the manufacturer’s instructions. Unsecured cannulas lacking transparent dressing or TA were used as controls (*n* = 3).

A 10-μL aliquot of a *S. epidermidis* RB02 suspension was applied at the edge of the TA or transparent dressing, or the dressing only for the combined TA and transparent dressing tests. Control plates had the bacteria seeded on the cannula entry point. The bacterial suspension was allowed to dry and was incubated overnight at 37 °C. The plates were scored for bacterial growth every 24 h up to 72 h. The presence of bacterial growth was determined by pH indicator dye which changes colour from purple (pH 6.8) to yellow (pH 5.2) when the bacteria ferment glucose in the media and produce acid.

### Chemical compatibility tests

To test chemical compatibility of TA and TA removal agent with ECMO cannulas, 23F Bio-Medicus femoral (Medtronic Inc., Minneapolis, MN) venous cannulas were cut into 15-cm lengths. The central 1-cm cannula section was exposed to Histoacryl or Remove (Smith & Nephew, Mississauga, ON) adhesive remover wipe per manufacturer instructions for 15 or 60 min (*n* = 6 per group per exposure time). All cannula sections were then subjected to mechanical testing to measure tensile strength; this was performed using an Instron 5567 (Instron Pty Ltd., High Wycombe, UK) universal testing machine at a crosshead speed of 250 mm/min. The force (N) needed to produce breakage of the cannula was recorded for every test of each group.

### Cannula securement (insertion site) tests

**Fig. 1:**
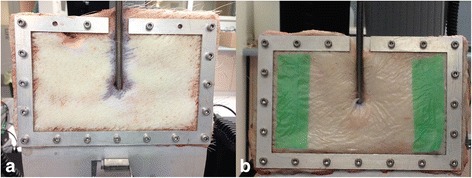
Simulated ECMO cannula insertion site. Cannula secured with **a** tissue adhesive, Histoacryl (*n* = 6), and **b** transparent dressing, Opsite (*n* = 6)

Adult porcine skin for testing of securement methods was obtained immediately following euthanasia of the animal. Skin sections measuring 13 cm × 20 cm were cut from a standard area of the animal then washed and dried with paper towel followed by hair removal using a surgical clipper. For each test, the skin section was attached by the edges to a purpose-built metal frame. A simulated ECMO cannula site was created using a modified Seldinger technique; a small incision was made in the centre of the skin section using a scalpel blade, and then, a 15-cm section of a Bio-Medicus Femoral 21F venous cannula was inserted through the incision following serial dilatation, allowing the hole to accommodate the cannula whilst ensuring a tight fit. For the TA test group, the cannula was secured with 0.5 mL of Histoacryl placed around the cannula insertion point and along the cannula to the end of the skin section (~ 7 cm), and allowed to dry for 30 min (Fig. 1a). For the transparent dressing test group, non-glued cannulas were covered with a single layer of Opsite cut to size (10 cm × 17 cm) and left untouched for 5 min after application (Fig. 1b). Six tests were performed for each group. In each test case, the metal frame containing the simulated cannula site was mounted onto the tensile testing machine (Instron 5567) and the pull-out force was measured (tensile force required to produce bond failure under the same conditions as described in ‘Chemical compatibility tests’).

### Tubing securement tests

**Fig. 2:**
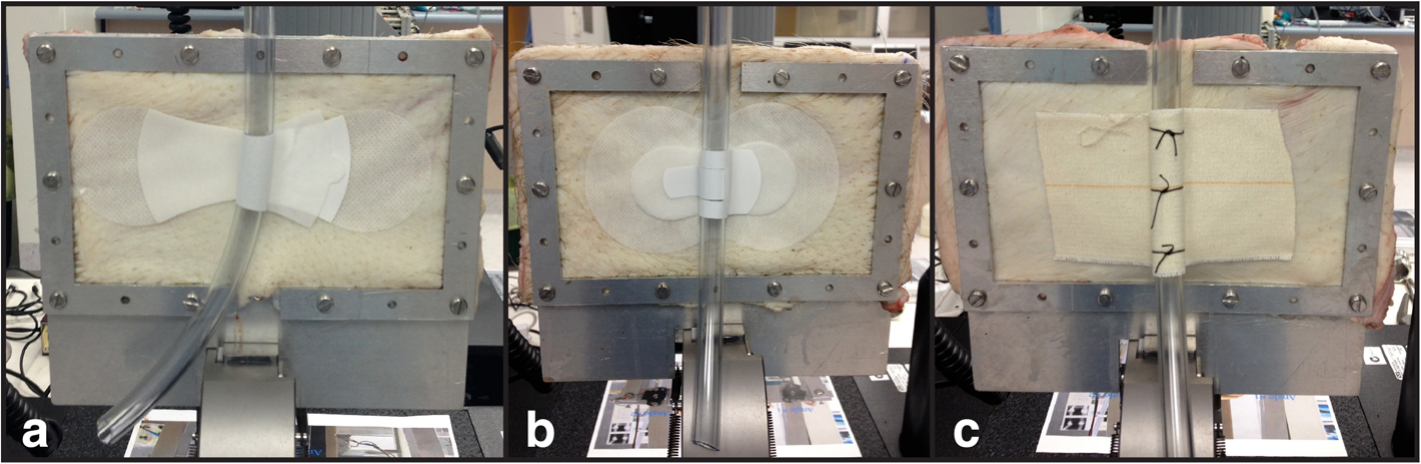
Skin with ECMO tubing attached and secured. **a** Grip-lok (*n* = 6). **b** MultiFix (*n* = 6). **c** Tensoplast adhesive bandage (*n* = 6)

Circuit tubing tests were undertaken using porcine skin sections prepared, framed and mounted according to the same method as described above. A simulated ECMO line securement point was created using 20-cm lengths of 3/8-in.-diameter tubing (Medtronic Inc., Minneapolis, MN) secured to the skin in three different groups (*n* = 6 for each group). Group 1 was secured with large-sized Grip-lok Universal Securement Device (TIDI Products, Neenah, WI) (Fig. 2a); group 2 was secured with a large-sized MultiFix UniFix Universal Tube and Catheter Securement Band (Midmed Pty Ltd., Murrarie, QLD, Australia) (Fig. 2b); and group 3 was secured to the skin with Tensoplast (BSN Medical, Mt. Waverley, VIC, Australia) elastic adhesive bandage using the method employed at our institution (Fig. 2c): Friars’ Balsam (LCM Ltd., Huddersfield, UK) was wiped onto the skin using a gauze pad and allowed to dry (to make the skin ‘tacky’ and improve adhesion of bandage to the skin); the elastic bandage was stuck onto the skin (along one side of the tubing), looped around the full circumference of the tubing, then stuck to the skin on the opposite side of the looped tape; the bandage was then anchored around the tubing using sutures placed through and around the tape loop (avoiding suturing of the skin) at three points along the tape section to prevent the bandage lifting away from the tubing.

#### Statistical analysis

All statistical analyses were performed using Prism 6.0 (Graphpad Software Inc.). Sample size calculations were based on similar testing previously reported [25]. Fisher’s exact test was used to statistically evaluate differences in bacterial growth observed between the transparent dressing and TA groups (microbiological tests). All data from mechanical testing (chemical campatibility, cannula securement, and tubing securement tests) were analysed using one-way ANOVA, followed by Tukey’s multiple comparison test or *t* test as appropriate. Statistical significance was defined as *p* < 0.05.

## Results

### Microbiological tests

**Fig. 3:**
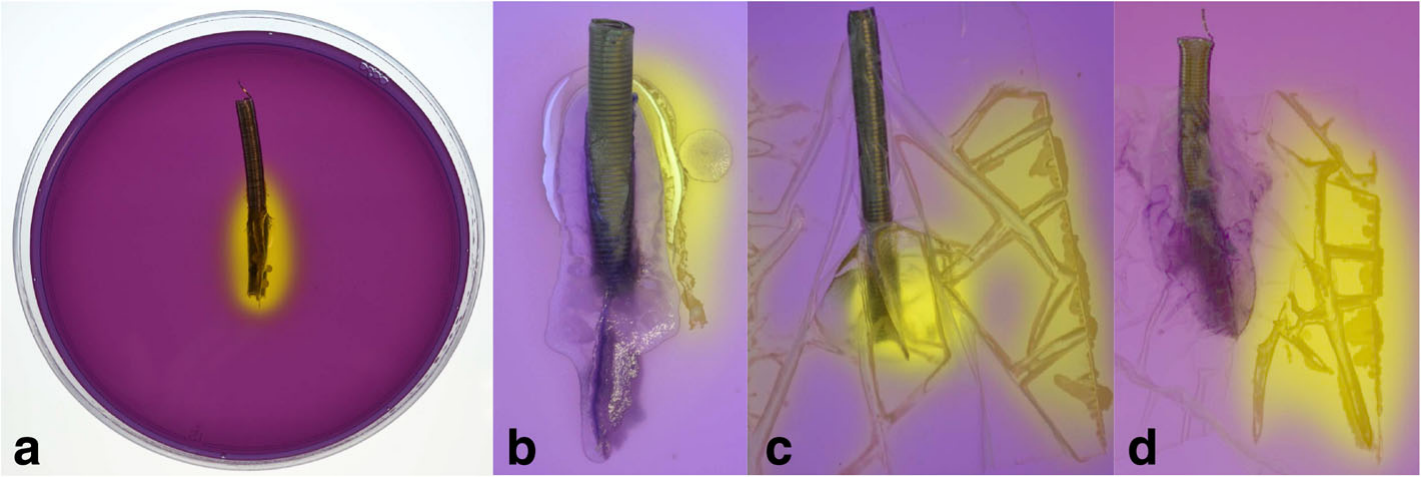
Microbiological test results. **a** Control plate: *S. epidermidis* growth on pH indicator agar. **b** TA applied at insertion site: TA inhibits *S. epidermidis* growth along the TA edge to the insertion point. **c** Transparent dressing applied over insertion site: *S. epidermidis* growth under the dressing, at the cannula insertion point and along the cannula dressing tunnel. **d** TA + transparent dressing: *S. epidermidis* growth under the dressing but inhibited beyond the TA edge. TA, tissue adhesive

At 24 h post-inoculation, bacterial growth was not observed in any TA test plates (Fig. 3b) whereas bacterial growth was present under the dressing in all transparent dressing test plates (Fig. 3c) (*p* = 0.002). TA also prevented penetration of bacteria at the cannula insertion point and along the tunnel (in agar) in all tests, when compared with control plates (*p* = 0.019). Bacterial migration to the insertion point did not differ between control and dressing plates with transparent dressing offering no protection from this occurrence (*p* = 0.167). In the combined dressing and TA test plates (Fig. 3d), TA inhibited migration of bacterial growth to the cannula insertion point and along the tunnel with bacterial growth observed only under the transparent dressing. Table 1 indicates the presence or absence of bacterial growth after 72 h of observation.

**Table 1:** *S. epidermidis* growth surrounding ECMO cannulas and securement product at 72 h post-inoculation

Securement product	Edge of securement product	Underneath securement product	Cannula insertion point	Along cannula tunnel
Histoacryl (*n* = 6)	+	−	−	−
Opsite (*n* = 6)	+	+	+	+
Histoacryl and Opsite (*n* = 6)	− (Histoacryl) + (Opsite)	− (Histoacryl) + (Opsite)	−	−
Unsecured (*n* = 3)	N/A	N/A	+	+

“+” growth at 72 h, “−” absence of growth at 72 h

### Chemical compatibility tests

Compared with control, cannula tensile strength was not reduced with the TA (132 vs 128 N) or TA remover agent (132 vs 134 N) at the 15-min exposure time (*p* = 0.57). However, increasing the TA remover agent exposure time to 1 h significantly weakened the cannula (132 vs 87 N, *p* < 0.01).

### Cannula securement (insertion site) tests and tubing securement tests

**Fig. 4:**
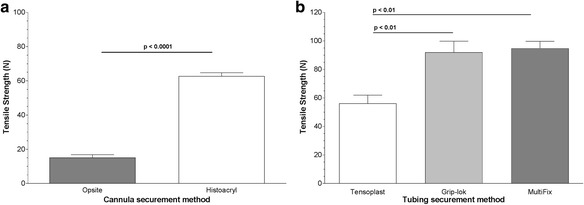
Mechanical test results. **a** Cannula securement comparing transparent dressing (Opsite) with TA (Histoacryl). **b** Tubing securement comparing adhesive bandage (Tensoplast) with SSD (Grip-lok and MultiFix). TA, tissue adhesive; SSD, sutureless securement device

TA significantly increased the pull-out force required to remove the cannula from the skin when compared with transparent dressing (62.8 vs 15.2 N, *p* < 0.0001; Fig. 4a). The force required to remove the tubing from the skin with both Grip-lok device (92.0 N) and MultiFix device (94.8 N) was very similar and was significantly increased compared with Tensoplast adhesive bandage (56.2 N) (*p* < 0.01; Fig. 4b).

## Discussion

This in vitro study demonstrated that TA is effective in providing a barrier for the prevention of bacterial migration to the ECMO cannula insertion site; TA significantly increases the force required to dislodge the cannula from the insertion site and, when used in combination with a SSD securing the circuit tubing, could substantially reduce the risk of accidental cannula movement and dislodgement. We found that TA prevented bacterial migration to the site of insertion and along the cannula tunnel, alone or in combination with transparent dressing. Additionally, TA was shown to be a superior option for ECMO cannula securement when compared to transparent dressing, which is commonly used to dress and secure cannulas at the point of percutaneous insertion. We established that the two commercial SSDs were more efficacious than the adhesive bandage method (Tensoplast) used at our institution to secure ECMO circuit tubing. Our findings suggest that TA may be useful in the bedside management of ECMO lines, both to prevent or minimise the potential for infection and to improve sutureless line securement.

To our knowledge, TA has not been tested as a bacterial inhibitor with ECMO cannulas. The complete capacity of TA to prevent bacterial migration in this in vitro study has significant potential in the clinical setting. When used to seal the ECMO cannula insertion site, TA effects may reduce extraluminal contamination via a bactericidal effect or a physical barrier effect on bacterial translocation at the skin puncture [29]. Therefore, TA has the potential to reduce localised and bloodstream infection in ECMO, and subsequently may decrease ICU stays, hospital stays and healthcare costs.

Our results also showed that neither TA nor TA remover wipe (Remove wipes) applied for 15 min weakened the cannula. However, exposure to TA remover wipe weakened the cannula after 60 min; therefore, all residual products must be removed from the ECMO cannula after TA removal. Avoiding prolonged contact and thoroughly wiping the area with normal saline to remove TA remover wipe residue must be key elements of cannula site care protocols. The manufacturer’s instructions regarding the removal of TA (Histoacryl) state that acetone may be used to remove residual product; however, this was not tested in this study as the investigators felt it would be inappropriate to use acetone on the cannulation site.

In testing the securement properties of TA at the site of cannula insertion, we found TA to be significantly stronger than transparent dressing. Other research shows TA to be a promising novel solution for intravascular device securement [18, 20, 23, 24]. Compared with transparent dressing, TA may have benefits in reduced ECMO cannula migration, as well as reduced infection risk by limiting pistoning or micromotion of the cannula and subsequent introduction of bacteria [17, 18]. Its clinical application would be advantageous for femoral cannulas given that peripheral cannulation for ECMO commonly involves the femoral vessels [31] and this location is a greater risk factor for colonisation and/or bloodstream infection compared with internal jugular sites in adults [32]. With regard to securement of the ECMO tubing, both of the commercial SSDs tested were significantly stronger than the method using adhesive bandage (Tensoplast). The greater tensile strength and therefore ability to limit gross line movement suggests SSDs could offer a safer clinical approach to reducing the risk of acutely life-threatening or fatal outcomes of accidental decannulation [33, 34]. SSDs have undergone limited clinical testing, but they appear safe, feasible and acceptable for central catheters [20]. The benefits of SSDs and TA in ECMO are summarised in Table 2.

**Table 2:** Tissue adhesive and sutureless securement device testing and clinical practice points for use in ECMO

Securement product	In vitro study findings	Advantages in ECMO	Clinical practice points
Cyanoacrylate tissue adhesive (Histoacryl)	• Higher tensile strength compared with transparent dressing at the cannula insertion point • Microbial inhibition along the cannula tunnel, under the cannula dressing and at the cannula insertion point compared with transparent dressing • Chemical compatibility of cannula and tissue adhesive remover agent (Remove wipes) after 15 min exposure, but cannula weakening observed after 1 h exposure time	• Promising simple, adjunct securement method to help stabilise peripheral cannulas at their percutaneous insertion point without the need for suturing • No risk for needlestick injury by avoiding cannula suturing • Potential to reduce incidence of cannula colonisation, localised infection and bloodstream infection	• Quick and easy to apply • Can remain in situ for days and be ‘topped up’ if needed • Easily removed with adhesive remover • Remover agent must be thoroughly cleansed from cannula after TA removal and long exposure times avoided • Prevents complications and failure in intravascular devices [39] • Low incidence of adverse skin effects such as skin tears has been reported [18, 22, 23] • Haemostatic properties prevent early post-insertion bleeding [40, 41] and early dressing change in patients with other intravascular devices [39] • Hair growth ‘against’ and ‘into’ TA with resultant pain on TA and central catheter removal has been reported [20]; hair must be clipped before applying TA at cannulation sites; rapid beard or pubic hair growth may preclude TA use • Further evidence required to guide clinical usage including the potential to reduce post-insertion cannula site bleeding in ECMO
Sutureless securement device (Grip-lok, MultiFix)	• Higher tensile strength compared with adhesive bandage (Tensoplast)	• More flexible approach and ease of use over adhesive bandages for securing lines • Avoids skin suturing and subsequent risk of oozing/bleeding and incidental perforation of tubing • Allows tubing to be easily readjusted or tightened as needed; therefore, less line handling needed and less risk of inadvertent line kinking or movement • When combined with TA, offers an optimal dual line securement strategy by minimising cannula micromotion (at insertion point) and gross movement of the tubing	• Simple and quick to apply • Velcro strap secures the hub of the device allowing for easy opening and closing • Can generally remain in place for several days and only needs changing when soiled • Not considered appropriate for securement of central ECMO cannulas in the absence of sutures but may help avoid gross line movement

The current study suggests that TA is a promising, simple alternative to traditional dressing methods and suturing which is itself associated with infection risk. We propose that the combined use of TA at the insertion site with SSDs along the ECMO tubing provides a feasible line management strategy which can be easily implemented at the bedside to prevent complications. Our findings have particular relevance to centres performing peripheral cannulation by percutaneous Seldinger technique since a key rationale of this practice is to avoid skin suturing and reduce insertion site bleeding [16, 35]. Our research highlights the importance of further investigation into the most effective methods for sutureless securement in this context, especially with the trend to increase patient mobility on ECMO [36, 37]; the need to transfer these patients for scanning or procedures; and the preference for multiport drainage cannulas which can rapidly entrain air through side holes should outward migration from the vessel occur [38]. A targeted research program investigating dressing and securement options is vital in providing valuable evidence to guide global practice in this area.

There were limitations to this study. Firstly, we tested only *S. epidermidis* due to its association with bloodstream infection during ECMO and its reported association with culture confirmed ECMO cannula infection [4]. We tested *S. aureus* in a previous laboratory study with the same results as *S. epidermidis* [25]. Secondly, microbiological and mechanical testing was performed using only one type of TA (butyl-cyanoacrylate) as our previous testing [25] demonstrated its superiority over octyl-cyanoacrylate. Thirdly, mechanical testing of the cannulas and tubing was performed separately rather than together as one continuous ECMO line. There are limitations in testing two mechanical systems in series, due to different strengths, inevitably that the load will not be shared evenly between the two sites, meaning one will always fail before the other at a similar failure force of that part on its own. Lastly, the use of TA to secure ECMO cannulas would not be appropriate for central ECMO cannulations therefore our findings could only be generalisable to peripheral cannulations.

## Conclusions

TA appears promising as a simple infection prevention and adjunct securement method for ECMO lines. Moreover, the combined use of TA at the cannula entry point with a sutureless device for tubing securement could provide an optimal overall strategy for line stabilisation, with the potential to reduce ECMO line-associated complications. A sufficiently powered randomised controlled trial will determine if the pre-clinical findings reported here translate into an effective strategy to reduce cannula-associated infection and cannula malposition or dislodgement.

## Abbreviations

ECMO: Extracorporeal membrane oxygenation
SSD: Sutureless securement device
TA: Tissue adhesive
